# Revisión de la infección oculta por el virus de la hepatitis B

**DOI:** 10.1515/almed-2021-0084

**Published:** 2022-08-15

**Authors:** Marta Lalana Garcés, Oihana Ortiz Pastor, Gemma Solé Enrech, Armando Raul Guerra-Ruiz, Gregori Casals Mercadal, Alejandro Almería Lafuente, María Antonieta Ballesteros Vizoso, Pablo Gabriel Medina, Sergio Salgüero Fernández, Angielys Zamora Trillo, Isabel Aured de la Serna, Juan Carlos Hurtado, Sofía Pérez-Del-Pulgar, Xavier Forns, Manuel Morales Ruiz

**Affiliations:** Comisión de Valoración Bioquímica de la Enfermedad Hepática, Sociedad Española de Medicina de Laboratorio (SEQC-ML), Barcelona, España; Servicio de Análisis Clínicos, Hospital de Barbastro, Huesca, España; Servicio de Bioquímica Clínica, Hospital Universitario Miguel Servet, Zaragoza, España; Servei de laboratori, UDIAT-CD. Corporació Sanitaria Parc Taulí, Sabadell, España; Servicio de Análisis Clínicos, Hospital Universitario Marqués de Valdecilla, Santander, España; Servicio de Bioquímica y Genética Molecular, CDB, Hospital Clínic de Barcelona, IDIBAPS, CIBEREHD, Barcelona, España; Servicio de Bioquímica Clínica, Hospital Royo Villanova, Zaragoza, España; Servicio de Análisis Clínicos, Hospital Universitario Son Espases, Palma de Mallorca, España; Servicio de Bioquímica Clínica, Hospital Universitari Vall d’Hebron, Barcelona, España; Servicio de Análisis Clínicos, Hospital Universitario Fundación Alcorcón, Madrid, España; Servicio de Bioquímica Clínica, Hospital General Universitario Gregorio Marañón, Madrid, España; Servicio de Digestivo, Hospital de Barbastro, Huesca, España; Servicio de Microbiología, CDB, Hospital Clínic de Barcelona, Universitat de Barcelona, Barcelona, España; Instituto de Salud Global de Barcelona (ISGlobal), Barcelona, España; Servicio de Hepatología, Hospital Clínic de Barcelona, IDIBAPS, CIBEREHD, Barcelona, España; Departamento de Biomedicina de la Facultad de Medicina y Ciencias de la Salud-Universidad de Barcelona, Barcelona, España

**Keywords:** ADN circular covalentemente cerrado (ADN-ccc), antígeno de superficie del virus de la hepatitis B ultrasensible (HBsAg ultrasensible), infección oculta por el virus de la hepatitis B (OBI)

## Abstract

**Introducción:**

El diagnóstico actual del virus de la hepatitis B (VHB) está basado en la detección mediante técnicas moleculares de ADN de VHB y ensayos serológicos, como el antígeno de superficie (HBsAg) y anticuerpos frente al core VHB (anti-HBc). Existe un grupo de pacientes con infección oculta de VHB (OBI) en los que estos ensayos no son capaces de detectar el HBsAg ni la cuantificación de ADN de VHB en sangre, aunque exista replicación activa en hígado.

**Contenido:**

El documento define la OBI, y los métodos actuales para su diagnóstico. También aborda la detección de pacientes con factores de riesgo y la necesidad de realizar el cribado de OBI en ellos.

**Resumen:**

Un correcto diagnóstico de OBI, previene la reactivación del VHB y su transmisión. El diagnóstico de OBI actualmente está basado en la detección de ADN de VHB en pacientes con HBsAg indetectable en sangre.

**Perspectivas:**

Un número elevado de pacientes con OBI puede permanecer sin diagnosticar. Es importante realizar el cribado de OBI en determinados pacientes con factores de riesgo. La introducción de nuevos marcadores, como el HBsAg ultrasensible, y estudios más profundos de marcadores, como el ADNccc hepático, serán necesarios para un correcto diagnóstico de OBI.

## Definición de infección oculta por el virus de la hepatitis b (VHB)

La infección oculta por VHB (occult HBV infection, OBI) se define como la presencia de ADN de VHB detectable en hígado y/o en suero en pacientes con antígeno de superficie (HBsAg) negativo.

La carga de ADN de VHB cuando se detecta en suero, es muy baja: <200 UI/mL (1,000 copias/mL) [1]. Algunos estudios mostraron que más del 90% de pacientes con OBI, presentaron carga viral en suero todavía más baja, alrededor de 20 UI/mL [2]. Otros estudios encontraron que el ADN puede ser detectado de manera intermitente en suero o plasma [3]. Además, estos pacientes suelen presentar marcadores bioquímicos hepáticos, como la ALT (alaninoaminotransferasa), con valores dentro de la normalidad [2].

Según el patrón serológico la OBI puede clasificarse en: 1/OBI seropositiva: presenta anticuerpos frente al core VHB (anti-HBc) y/o anticuerpos frente al antígeno de superficie (anti-HBs) positivos y 2/OBI seronegativa: con anti-HBc y anti-HBs negativos [1]. Todos ellos presentan ADN de VHB detectable en tejido hepático (Tabla 1) [4].

**Tabla 1:** Marcadores en suero e hígado en pacientes OBI.

	Suero		Hígado	
	Anti-HBc	Anti-HBs	ADN de VHB	ADN de VHB
OBI seropositivos	+	+	<200 UI/mL/no detectable	+
OBI seronegativos	–	–	<200 UI/mL/no detectable	+

OBI, infección oculta de virus de la hepatitis B.

En pacientes con OBI seropositiva, el HBsAg se vuelve indetectable siguiendo la resolución de la hepatitis aguda, después de pocos meses o después de años si se trata de una infección crónica. Desde el punto de vista inmunológico se desconoce si los pacientes con infección o enfermedad crónica por VHB que pierden el HBsAg con la terapia antiviral, son comparables con aquellos pacientes que aclaran de forma espontánea el HBsAg. Las posibles implicaciones de esta distinción todavía se desconocen [3].

La cantidad de pacientes seronegativos varía entre el 1% y el 22% de pacientes con OBI [5, 6]. Estos pacientes podrían haber perdido progresivamente los anticuerpos frente a VHB (anti-HBc y anti-HBs) o haber sido negativos desde el comienzo de la infección [3].

**Figura 1:**
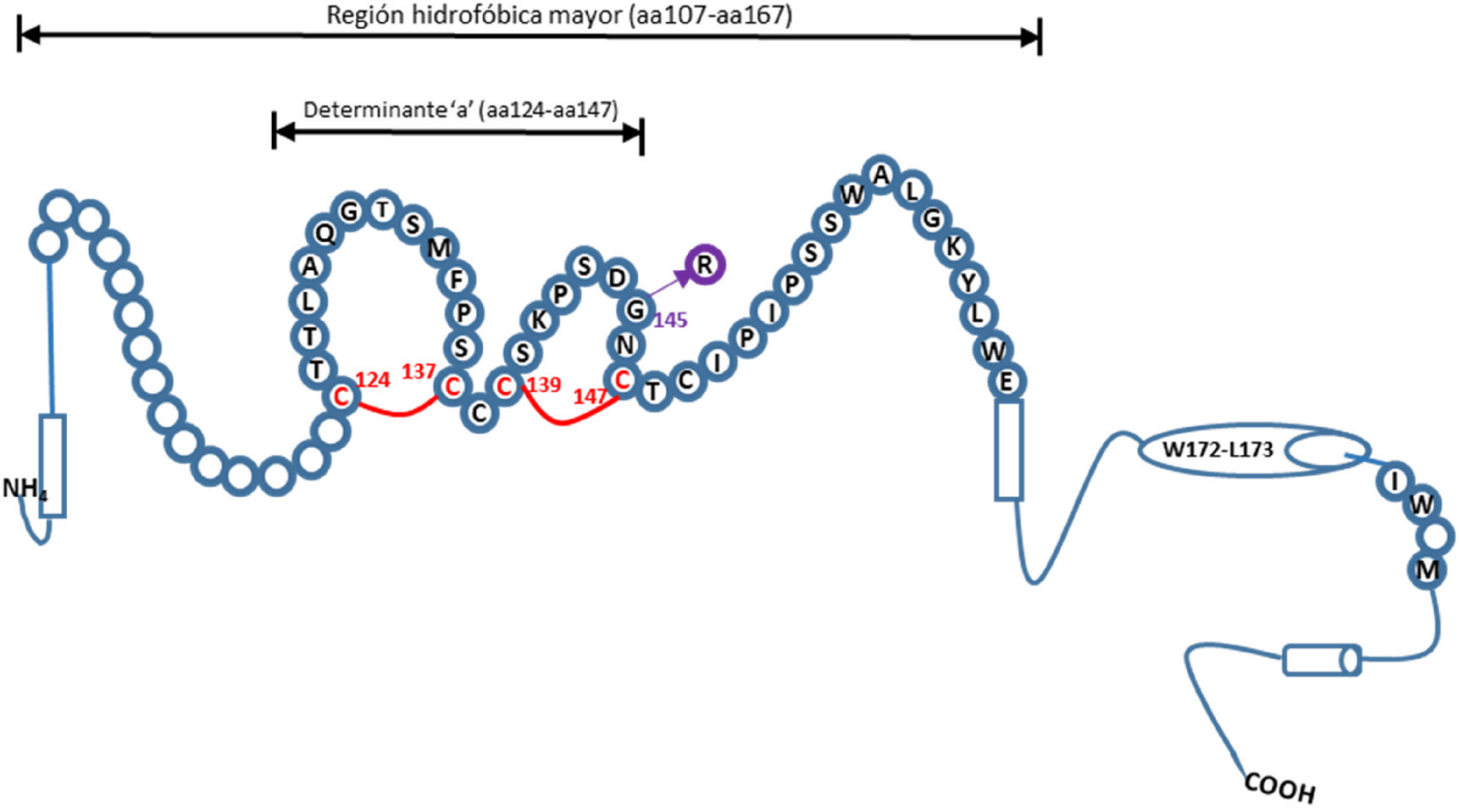
Estructura secundaria del HBsAg. La región hidrofóbica mayor (aminoácidos 107 a 167) y el determinante “a” (aminoácidos 124 a 147) están representados con círculos con el nombre del respectivo aminoácido (código de una letra). El determinante “a” es el epítope inmunodominante, el cual se mantiene mediante dos puentes disulfuro *C124–C137* y *C139–C147*. En violeta se muestra una de las mutaciones más comunes. Adaptado de Rios-Ocampo y col. y Jaramillo y col. [4, 8].

Además, existe un subgrupo de pacientes con OBI denominados “falsos OBI”, debido a que son portadores de mutaciones en el HBsAg que no son detectadas por algunos ensayos utilizados en laboratorios de rutina y presentan niveles de ADN de VHB más elevados, comparables a los que normalmente se detectan en infecciones por VHB evidentes, con HBsAg positivo [1, 7]. Estos pacientes son infectados con variantes que portan mutaciones en el gen S, denominadas variantes de escape. Se trata de mutaciones en la secuencia que codifica el determinante “a” (Figura 1) [4, 8]. En el segundo bucle del determinante “a” se ha encontrado la mutación más común y mejor caracterizada (cambio de glicina por arginina en la posición 15 [G145R]). Esta mutación fue descrita por primera vez por Carman y colaboradores, en un niño nacido de madre portadora que había sido vacunado y tratado con inmunoglobulinas específicas tras el parto, y a pesar de ello presentó infección por VHB [9], [10], [11].

Las variantes de escape plantean un riesgo importante. Por un lado, la detección del HBsAg por algunos inmunoanálisis, puede dar lugar a resultados falsos negativos, es decir, no son reconocidos por algunos ensayos comerciales. Por otro lado, las actuales vacunas frente al VHB pueden no ser efectivas en la infección debido a estas mutaciones [9], [10], [11].

Otras mutaciones que pueden dar lugar a “falsas OBI” son las que se observan frecuentemente en las regiones preS1/preS2. Las mutaciones en estos promotores de proteínas de superficie muestran alteraciones en la expresión del HBsAg. Estos cambios hacen que disminuyan significativamente los niveles de HBsAg o incluso que sean indetectables [12].

Por último, los “*splicing steps”* tienen un efecto crítico en la expresión genética del VHB. La sustitución de Guanina por Adenina en la posición 458 del gen S del ARN mensajero interfiere en el paso de *splicing* y ha sido asociado con la ausencia de la expresión del HBAgs y baja replicación de ADN de VHB [13].

## Curso de la infección por VHB y reactivación

El VHB se trasmite por vía perinatal, parenteral, sexual y potencialmente mediante contacto de persona a persona [14]. En la fase aguda y ante la presencia del virus, el sistema inmune se activa produciendo la destrucción de hepatocitos infectados. Si la infección ocurre en recién nacidos y niños, los cuales presentan un estado de inmunotolerancia, la evolución a cronicidad es muy elevada, se estima un 90% en recién nacidos y un 30% en niños, en ausencia de inmunoprofilaxis adecuada [14, 15]. Por otra parte, la infección en jóvenes y adultos presenta una fuerte respuesta inmune.

La curación clínica depende de la interacción entre la respuesta inmune y la actividad replicativa del virus. En la mayoría de los casos cuando los mecanismos inmunomoduladores son eficaces, la primoinfección se resuelve sin síntomas específicos. Por ello en adultos, más del 95% de las infecciones por VHB son autolimitadas [14]. En el 5–10% [15] de los casos la inmunomodulación excesiva provoca una respuesta inflamatoria severa en el hígado provocando síntomas de enfermedad aguda y aumento importante de la concentración de aminotransferasas, que en la mayoría de los casos se resuelve en un tiempo inferior a 6 meses. Excepcionalmente, en menos del 1% de los casos, esta respuesta inmune puede provocar una lisis rápida y masiva de los hepatocitos infectados dando lugar a un fracaso hepático fulminante. Por último, en un 5–10% de los casos el sistema inmune es incapaz de controlar la replicación viral y la infección se vuelve crónica [15].

**Figura 2:**
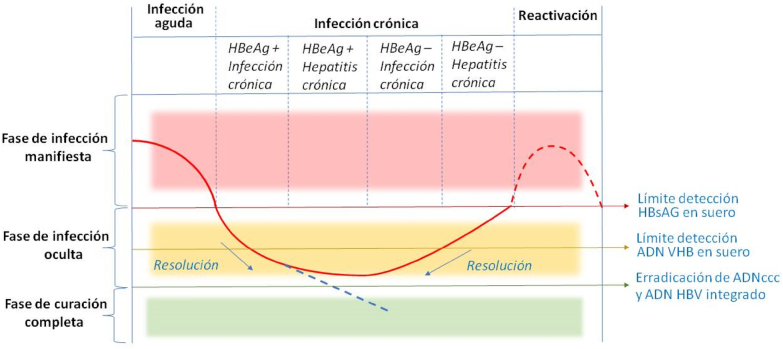
Fases de persistencia y reactivación de VHB*.* La curva roja continua indica evolución de la actividad de replicación viral en infecciones agudas, crónicas y ocultas. La línea azul de puntos indica la fase hipotética de curación completa. La curva roja de puntos representa la actividad de replicación viral en la fase de reactivación. HBeAg, antígeno e de la hepatitis B; HBsAg, antígeno de superficie de la hepatitis B; ADNccc, ADN circular covalentemente cerrado (modificada a partir de Shi y col.) [16].

La hepatitis B crónica es una enfermedad dinámica, con diferentes fases clínicas no consecutivas en el tiempo. La Figura 2 [16] muestra el curso de la infección por VHB que se clasifica en 4 fases y está basada en marcadores bioquímicos, serológicos e histológicos. Estas fases están clasificadas en función de la positividad o negatividad del HBeAg, de la carga viral (ADN de VHB), de la concentración de ALT y eventualmente del grado fibrosis hepática (FH) (Tabla 2). Es necesario realizar determinaciones seriadas de estas magnitudes para clasificar correctamente a los pacientes. La EASL (*European Association for the Study of the Liver)* denominó en el 2017 las diferentes fases asignándoles una nueva nomenclatura tal y como se detalla a continuación [17]:

– **Fase 1: Infección crónica HBeAg positivo (fase de inmunotolerancia).** Fase caracterizada por la presencia de HBeAg sérico, niveles muy elevados de ADN de VHB y niveles de ALT dentro del rango de normalidad de manera persistente. En el hígado la inflamación y la fibrosis son mínimas o nulas. Sin embargo, el elevado nivel del ADN de VHB sugiere que la hepatocarcinogénesis ya podría estar en curso en esta fase temprana de la infección. Es la fase más frecuente en sujetos infectados perinatalmente y puede prolongarse hasta pasados 10–30 años. Estos pacientes son muy contagiosos debido a los altos niveles de ADN de VHB.
– **Fase 2: Hepatitis crónica HBeAg positivo.** Se caracteriza por la presencia de HBeAg en suero, y niveles elevados de ADN de VHB y de ALT. En el hígado se observa necroinflamación moderada o grave y progresión acelerada de la fibrosis. Es más frecuente en individuos infectados durante la edad adulta, aunque también puede ocurrir tras varios años en fase de inmunotolerancia. La mayoría de los pacientes pueden lograr la seroconversión del HBeAg a anti-HBe y supresión del ADN de VHB y entran en la fase de infección HBeAg negativa.
–
**Fase 3: Infección crónica HBeAg negativo (fase portador inactivo).** Se caracteriza por la presencia de anticuerpos frente al HBeAg (anti-HBe), niveles de ADN VHB muy bajos o indetectables (<2.000 UI/mL) y ALT dentro del rango de normalidad. Sin embargo, algunos pacientes en esta fase pueden tener niveles de ADN de VHB por encima de 2.000 UI/mL (normalmente <20.000 UI/mL) acompañados de niveles normales persistentes de ALT con mínima inflamación hepática y bajo grado de fibrosis. Si permanecen en esta fase estos pacientes presentan bajo riesgo de progresión a cirrosis o a carcinoma hepatocelular (CHC), pero pueden evolucionar a hepatitis crónica. La pérdida de HBsAg o seroconversión a anti-HBs puede ocurrir de manera espontánea en un 1–3% anual de los casos. Generalmente presentan niveles bajos en suero de HBsAg.
–
**Fase 4: Hepatitis crónica HBeAg negativo.** Se caracteriza por falta de HBeAg sérico, generalmente con anti-HBe detectable, niveles fluctuantes moderados o elevados de ADN de VHB en suero (a menudo más bajos que en pacientes HBeAg positivos) y valores de ALT aumentados. La histología del hígado muestra inflamación y fibrosis. Esta fase se asocia con bajas tasas de remisión espontánea de la enfermedad.
–
**Infección oculta por VHB.** Como se ha comentado al inicio, la definición de OBI se caracteriza por HBsAg negativo, con o sin anticuerpos anti-HBc o anti-HBs. Los pacientes presentan valores normales de ALT y generalmente, aunque no siempre, niveles indetectables de ADN de VHB en suero. El hígado presenta frecuentemente niveles detectables de ADN circular covalentemente cerrado (ADNccc). Si la pérdida del HBsAg ha ocurrido antes del inicio de la cirrosis, se asocia con mínimo riesgo de cirrosis, descompensación y CHC, y supone una mayor supervivencia. Sin embargo, si la pérdida del HBsAg se produce en un paciente cirrótico, se debe continuar la vigilancia, puesto que existe riesgo de CHC. La inmunosupresión puede provocar reactivación de VHB en estos pacientes [17].

**Tabla 2:** Clasificación de las fases crónicas del VHB.

Cronicidad VHB: HBsAg positivo (>6 meses)				
	HBeAg positivo		HBeAg negativo	
	Fase 1. Infección crónica (fase de inmunotolerancia)	Fase 2. Hepatitis crónica	Fase 3. Infección crónica (portador inactivo)	Fase 4. Hepatitis crónica
ADN VHB	>10^7^ U/mL	10^4^–10^7^ U/mL	<2000 UI/mL	>2000 UI/mL
ALT	Normal	Aumentada	Normal	Aumentada
FH	No fibrosis/mínima	Moderada/severa	No fibrosis	Moderada/severa

ALT, alanina aminotransferasa; FH, fibrosis hepática.

## Mecanismo molecular y respuesta inmunitaria

La eliminación del genoma del VHB no suele ser completa tras hepatitis agudas, incluso después de infecciones por VHB leves. Esto es debido a que algunos genomas del VHB permanecen como ADNccc de forma oculta en el hígado. Su expresión está controlada en gran medida por el sistema inmunológico del huésped [18].

El ADNccc es el molde de todos los transcritos virales y, por tanto, es capaz de generar partículas infecciosas. El ADN de VHB también puede integrarse en el genoma del huésped y permanecer en los hepatocitos de individuos infectados por VHB después del aclaramiento del HBsAg espontaneo o inducido por tratamiento. Sin embargo, este ADN de VHB integrado no presenta replicación competente, no produce viriones, pero puede producir HBsAg en forma de partículas subvirales, a través de una vía independiente [16].

La base molecular de la OBI está relacionada con la estabilidad y la persistencia a largo plazo del ADNccc en el núcleo de los hepatocitos infectados. El ADNccc episomal existe como un minicromosoma viral, similar a un plásmido el cual es muy estable y duradero [16]. Además, la elevada vida media de los hepatocitos, implica que la infección por VHB, puede continuar toda la vida [3]. La mayoría de casos de OBI presentan bajos niveles de ADNccc en hígado. La supresión de la actividad de replicación y la expresión de la proteína viral es regulada a través de la inmunidad del huésped y mecanismos epigenéticos. El bajo nivel de ADNccc que presenta transcripción activa da lugar a una baja o indetectable transcripción de ARN y, en consecuencia, baja traducción y expresión de proteínas.

La elevada prevalencia de la OBI en todo el mundo sugiere que el sistema inmunológico es eficaz para controlar el VHB, aunque no se elimine definitivamente, ya que el ADNccc persiste durante años.

La respuesta inmune antiviral es estimulada continuamente mediante bajas concentraciones intermitentes de antígenos del VHB [3]. Un pequeño número de casos de OBI son debidos a infección con mutantes de VHB con defectos en la actividad de replicación, debido a mutaciones en el promotor genómico de la proteína S. La OBI muestra respuesta inmune duradera y específica de células T frente a epítopos del VHB, con una respuesta más eficiente entre individuos OBI-seropositivos [1].

## Diagnóstico

El diagnóstico de OBI está basado en la detección de ADN de VHB en sangre o en hígado de individuos con HBsAg negativo. El método de referencia (*gold estándar*) es la detección de ADN de VHB en hígado, aunque al tratarse de un método sumamente invasivo, frecuentemente se realiza la detección de ADN de VHB en sangre. A menudo, se utiliza la detección de anti-HBc en sangre como sustitución a la detección de ADN de VHB. Aunque habría que tener en cuenta que en caso de OBI seronegativas estos anticuerpos serán negativos. Por tanto, el diagnóstico de OBI está basado en la detección de ADN de VHB y del HBsAg. Los ensayos de detección de HBsAg que presenten sensibilidad inadecuada para la detección de variantes S del VHB (*variantes de escape*) pueden conducir a resultados falsos negativos del HBsAg y a un diagnóstico erróneo de OBI en personas con infección evidente por el VHB. Por otra parte, las pruebas de detección de ADN de VHB con sensibilidad inadecuada pueden dar lugar a resultados falsos negativos y a la pérdida de diagnósticos de OBI.

El límite de detección del HBsAg de los ensayos comerciales que se utilizan actualmente es 50 mUI/mL. Estudios recientes han desarrollado kits de detección de antígenos con elevada sensibilidad (hasta 10 veces más sensibles), denominados ultra-sensibles, como el “Lumipulse G HBsAg-HQ”, un inmunoensayo enzimático mediante quimioluminiscencia que presenta un límite de detección de 5 mUI/mL [19, 20]. Otros estudios han mostrado que el ensayo ultra sensible de Abbot ARCHITECT con un límite de detección de 5,2 mUI/mL del HBsAg presentó un rendimiento clínico cercano al de detección de ADN de VHB [21].

Recientemente se han desarrollado ensayos para detección y cuantificación de HBsAg todavía más sensibles, con un límite de detección de 0,5 mUI/mL. Estos ensayos podrían ser utilizados en pacientes con VHB resuelta para identificar actividad transcripcional del ADNccc y replicación mínima del VHB y poder detectar OBI. Shinkai y colaboradores realizaron un estudio comparando 3 ensayos de detección de HBsAg con diferentes límites de detección (50, 5 y 0,5 mUI/mL) en muestras de suero de 120 pacientes con enfermedades hematológicas que estaban recibiendo quimioterapia sistémica. En 13 de ellos se detectó reactivación del VHB mediante la elevación del ADN de VHB. Los autores sugieren que la sensibilidad del ensayo semi-automático de transferencia de inmunocomplejos por quimioluminsicencia (ICT-CLEIA, Sysmex Corporation) para detectar HBsAg fue comparable a la cuantificación del ADN de VHB. El ensayo ICT-CLEIA fue capaz de detectar la presencia de HBsAg en 12 de los 13 pacientes que presentaron reactivación, en 2 de ellos incluso antes que la elevación del ADN de VHB. Por tanto, el ensayo de ICT-CLEIA se confirma como un nuevo ensayo de HBsAg ultrasensible para seguimiento del VHB y para prevenir la reactivación del VHB, así como la transmisión de OBI [22].

Además de la sensibilidad, los reactivos de HBsAg difieren en la capacidad para detectar variantes de escape en la región S. La identificación de las variantes del gen S es esencial para un correcto diagnóstico y para sus posibles implicaciones clínicas, por ello se recomienda la utilización de anticuerpos anti-HBs multivalentes en los ensayos para detección de HBsAg para una óptima detección de estas variantes. El uso de anticuerpos anti-HBs con múltiples epítopos del HBsAg debería ser obligatorio en los reactivos para garantizar la correcta detección del HBsAg.

Como se comentó anteriormente el método ideal para el diagnóstico de OBI es la detección de replicación de ADN de VHB en el hígado. Sin embargo, todavía no están disponibles ensayos estandarizados y validados para esta determinación. La detección del anti-HBc en sangre puede ser utilizada como un marcador sustituto para identificar OBI en personas que han recibido terapia inmunosupresora y en estudios epidemiológicos. El grupo de Taormina recomienda su utilización cuando la determinación de ADN de VHB no está disponible. Actualmente los ensayos de detección de anti-HBc presentan una especificidad bastante elevada (≥99%) [3].

## Prevalencia OBI

La prevalencia de OBI presenta elevada variabilidad a lo largo de todo el mundo. En regiones endémicas como Asia, esta prevalencia puede ser tan elevada que llegue a superar el 8% [2, 6].

La prevalencia descrita varía de 1% a un 87%, pero estos resultados deben ser interpretados con cautela [2]. Deben tenerse en cuenta los factores de riesgo en el grupo de pacientes estudiado, los problemas de muestreo, la sensibilidad del ensayo utilizado, así como la prevalencia de HBsAg en la región geográfica sometida a estudio [3].

La prevalencia de OBI parece ser mayor en individuos con factores de riesgo para infección por VHB: pacientes coinfectados con el virus de la hepatitis C (VHC) (52%) [23] o el virus de la inmunodeficiencia humana (VIH) (15%) [24], pacientes en hemodiálisis (2%) [25], o con cirrosis criptogénica (14%) [26].

El rendimiento y la sensibilidad de los ensayos de HBsAg y ADN de VHB, las características de la población estudiada, la prevalencia de infección con HBsAg positivo en la población general y los criterios utilizados para definir OBI pueden influir en la prevalencia de OBI. Por lo tanto, es difícil comparar datos en diferentes estudios y la prevalencia en la población general sigue siendo en gran medida indefinida debido a estas limitaciones [3].

## Factores de riesgo

La detección de factores de riesgo de OBI es importante para prevenir el riesgo de transmisión. Los principales factores de riesgo se muestran en la Tabla 3 [13].

**Tabla 3:** Factores de riesgo de OBI.

Factores de riesgo OBI
Pacientes con historia previa de infecciones por VHB
Pacientes coinfectados con el VHC o el VIH
Receptores de trasplante de órganos
Donantes de sangre
Donantes de órganos
Pacientes con talasemia o hemofilia
Pacientes con hepatitis criptogénica, cirrosis o CHC
Pacientes de hemodiálisis
Pacientes tratados con lamivudina o interferón
Niños en edad de vacunación, especialmente en áreas endémicas de VHB
Pacientes inmunodeprimidos por tratamientos biológicos u otros tratamientos de quimioterapia (asociada a tratamiento con anti-CD20)

## Pacientes coinfectados con VHC

La prevalencia de coinfección actual o previa por VHB en pacientes infectados por VHC es relativamente frecuente, debido a que el VHB y el VHC tienen vías de transmisión similares. Además, la OBI aparece con frecuencia en pacientes con infección crónica por VHC.

Estudios recientes muestran prevalencias variables de OBI entre pacientes con VHC que varían de 0 a 52% [23].

En un estudio realizado por Mandour y col. en la región del Canal de Suez, en el nordeste de Egipto se determinó la prevalencia de OBI en pacientes con insuficiencia renal crónica terminal en hemodiálisis y en pacientes con VHC crónica. La prevalencia de OBI fue mayor en los pacientes con VHC frente a los pacientes en hemodiálisis, con unas prevalencias de 8,5% y 1,8%, respectivamente [27]. Emara y col realizaron un estudio también en Egipto en 155 pacientes con VHC crónica en tratamiento con interferón pegilado/ribavirina. Se determinó una prevalencia de un 3.9% de OBI, y afectó a pacientes de menor edad, asociados con una carga viral basal del VHC más elevada y con menos fibrosis hepática que los pacientes monoinfectados [28]. En otro estudio realizado por Bal y Onlen en pacientes con infección crónica por VHC y tratados con interferón se determinó una prevalencia de OBI del 1% [29].

La prevalencia de OBI en pacientes coinfectados por VHC puede ser debida a varias causas: una de ellas podría ser las mutaciones observadas en el gen de HBsAg en estos pacientes [30]. Otra causa podría ser la baja replicación del VHB, ya que cuando coexisten el genoma del VHB y el de VHC en el mismo hepatocito, la replicación del VHB está inhibida debido a la interferencia de las moléculas de VHC [31].

El impacto clínico de la OBI en pacientes con VHC crónica es todavía desconocido. Algunos estudios sugieren que la presencia de OBI podría estar asociada con daño hepático más severo, cirrosis y mayor tasa de CHC. La OBI también se ha considerado causa de fracaso de la terapia con interferón en algunos estudios [32]. Otros en cambio no encuentran la OBI como causa significativa para la falta de respuesta a la terapia con interferón/ribavirina [28]. El tratamiento con antivirales de acción directa (AAD) puede aumentar la detección del ADN del VHB en pacientes con OBI, si bien parece ser un evento raro y sin consecuencias clínicas o virológicas, al menos en pacientes inmunocompetentes [33, 34].

Para mejorar tratamientos y consecuencias de la OBI, se recomienda realizar el cribado de anti-HBc y ADN-VHB antes del tratamiento de pacientes infectados por VHC [11]. El tratamiento con AAD se ha asociado con un mayor riesgo de reactivación del VHB en pacientes HBsAg positivo [35] en algunos casos con hepatitis graves. Las guías de manejo del VHB recomiendan, de hecho, profilaxis con análogos nucleós(t)idos en estos pacientes durante el tratamiento. Aunque también existen datos de reactivación del VHB en pacientes con OBI, parece que ésta se limitaría a pequeñas elevaciones del ADN de VHB sin repercusión clínica. No obstante, es recomendable verificar la presencia de HBsAg y anti-HBc en pacientes que van a iniciar tratamiento de VHC con AAD.

## Pacientes coinfectados con VIH

La coinfección de OBI y VIH ocurre principalmente, entre usuarios de drogas por vía parenteral, ya que el VHB y el VIH comparten la misma vía de transmisión, al igual que ocurre con la infección crónica por VHC.

La prevalencia de OBI entre pacientes infectados por VIH es incierta, puede variar desde 0% [36], hasta 15% [24] y las consecuencias clínicas de esta coinfección son poco conocidas. En un estudio retrospectivo realizado por Marquet-Juillet en 31 pacientes infectados por VIH se detectó ADN de VHB en 7 pacientes (22%). En este estudio la OBI parece ser más frecuente entre pacientes coinfectados además con VHC. Todos ellos presentaron anticuerpos anti-HBc. El número de células CD4+ fue significativamente menor en las muestras con ADN de VHB detectable. La prevalencia de OBI entre pacientes infectados por VIH y portadores aislados de anti-HBc fue elevada, con baja carga viral de VHB (<20 UI/mL) [37].

En África, en especial en la región Subsahariana, donde la transmisión de VIH y VHB es predominante por vía sexual, la prevalencia de OBI también es variable, con prevalencias de 5,3% y 18,7% en Kenia [38, 39], 5,9% en Camerún [40], 6,7% y 23% en Sudáfrica [41, 42], 15,1% en Sudán [43] y 26,5% en Bostwana [44]. También existe amplia variación entre diferentes regiones dentro de un mismo país como ocurre en Kenia y Sudáfrica.

La infección por VIH empeora el curso de la enfermedad crónica por VHB, puede conducir a una progresión más rápida de fibrosis hepática, desarrollo de cirrosis y CHC, una menor tasa de seroconversión espontánea del HBsAg y del HBeAg y mayor riesgo de reactivación de VHB en portadores inactivos. Por otro lado, el VHB no influye en el curso de la enfermedad por VIH [45]. La persistencia de VHB y VIH puede dar lugar a hepatitis severa e incluso fulminante. Por todo ello se recomienda realizar el cribado de anti-HBc y ADN de VHB antes de iniciar el tratamiento en pacientes con VIH [13].

## Donantes de sangre

La prevalencia de OBI en donantes de sangre es muy baja. En Europa la OBI se ha identificado en 1:1.000 a 1:50.000 donaciones de sangre [46]. Según datos actualizados se estima que el riesgo en nuestro país es de un caso por cada 170.000 [47]. En un estudio de donantes de sangre en un área altamente endémica de VHB, la prevalencia de OBI fue muy baja, del 0,11 y 0,13%, lo que implicó un impacto muy bajo sobre los servicios de transfusión [2]. Sin embargo, la incidencia de transmisión de VHB mediante transfusión de donantes con OBI podría estar subestimada.

Esto es debido a diversas causas: en la mayoría de los casos la infección por VHB cursa sin evidencia clínica de hepatitis aguda en los receptores. Por otra parte, los niveles de ADN de VHB son extremadamente bajos o detectables de manera intermitente. Por último, el volumen limitado de muestras archivadas de donantes, en ocasiones no permite determinar el ADN de VHB [3].

La detección de ADN de VHB mediante técnicas de amplificación de ácidos nucleicos (NAT) es más sensible que el ensayo de HBsAg como medida preventiva contra la transmisión de VHB mediante transfusión sanguínea [13]. La combinación de ambas pruebas, previene de manera eficiente la mayoría de transmisiones de VHB. Sin embargo, puede persistir un riesgo residual asociado con los niveles de ADN de VHB extremadamente bajos y la carga viral intermitente en sangre de donantes con OBI. Diversos estudios han reportado que la transmisión de VHB mediante transfusión de componentes de sangre desde donantes con OBI, contenían una baja carga viral (<200 viriones). Modelos basados en evidencias clínicas y experimentales estiman un riesgo de transmisión residual de 3–14% asociados con donaciones con OBI que presentan HBsAg y cargas virales VHB negativos [48]. La determinación del anti-HBc podría mejorar la seguridad de la sangre de donantes [49]. Además, la presencia de anti-HBs en los receptores reduce significativamente el riesgo de infección [46].

En España el Comité Científico de Seguridad Transfusional (CCST) estimó que el riesgo residual de transmisión del VHB es más elevado que para el VIH y para el VHC. En el año 2009 para disminuir la transmisión se propusieron dos opciones: el cribado de todas las donaciones para anti-HBc y el cribado para ADN de VHB. El primero, además de no detectar anticuerpos al inicio de la infección, supone (debido a su elevada prevalencia y baja especificidad), una pérdida del 4–5% de las donaciones, lo que desaconseja su implantación. La segunda opción, además de detectar la infección desde su inicio, presenta la ventaja de detectar infecciones por OBI con HBsAg indetectable. Por ello el CCST recomienda en España el cribado sistemático para VHB mediante determinación de ADN de VHB de toda donación de sangre mediante NAT. Además, señala que el cribado para ADN de VHB no puede considerarse sustituto de HBsAg [47].

## OBI en hemodiálisis

Los pacientes en hemodiálisis son pacientes en riesgo para la infección por VHB: son frecuentemente transfundidos, están inmunodeprimidos y sometidos a procedimientos invasivos con manipulación de sangre, y por ello deben ser estudiados serológicamente. Además, todos los pacientes seronegativos deben ser vacunados. En los últimos años la prevalencia de VHB en las unidades de hemodiálisis ha disminuido de manera importante hasta el 1,03% [50].

El diagnostico de daño hepático basado en los niveles de aminotransferasas es difícil, la uremia puede inhibir las reacciones inflamatorias en el hígado y por ello se atenúa la destrucción de los hepatocitos.

En un estudio realizado por Sowole y col en una cohorte multiétnica de pacientes en hemodiálisis en Londres, con anti-HBc positivo y HBsAg negativo, se determinó una prevalencia de OBI del 2,2%. Todos ellos presentaron niveles muy bajos de carga viral (<10 UI/mL), con bajo riesgo de transmisión nosocomial debido a la presencia de un sólido programa de vacunación activa frente al VHB. Una de las limitaciones del estudio fue la medición de la carga viral solo una vez, lo cual no excluye que estos pacientes tuvieran cargas más elevadas, debido a que en ocasiones la carga es intermitente [25].

La evaluación cuantitativa del ADN de VHB constituye el método más eficiente para evaluar la OBI en pacientes en hemodiálisis. Por ello se recomienda que los pacientes en hemodiálisis sean cribados de manera rutinaria de VHB, entre otros, y también de OBI, utilizando técnicas de elevada sensibilidad molecular para prevenir la transmisión nosocomial.

## OBI y enfermedad hepática criptogénica

La enfermedad hepática criptogénica se caracteriza por su etiología de origen desconocido y su prevalencia varía enormemente en diferentes regiones del mundo. La OBI se ha demostrado en pacientes con ALT persistentemente elevada sin otras causas aparentes y ha sido considerada como factor de riesgo adicional para la progresión a cirrosis y a CHC. Hashemi y col investigaron la prevalencia de OBI en un grupo de 50 pacientes con cirrosis criptogénica y 80 pacientes sanos como grupo control. En el grupo de pacientes con cirrosis criptogénica encontraron 7 (14%) pacientes con ADN de VHB positivo determinado mediante PCR. De los cuales, 4 de ellos (57%) fueron seronegativos, es decir presentaron marcadores anti-HBc y anti-HBs negativos. En el grupo control ningún paciente tuvo ADN de VHB positivo. El estudio concluye que la prevalencia de OBI es frecuente en pacientes con cirrosis criptogénica, especialmente en los de mayor edad. Esto puede contribuir a una rápida progresión a descompensación hepática, por lo que sugieren la investigación de OBI en pacientes con cirrosis hepática [26].

Para mejorar el tratamiento y manejo de pacientes con enfermedad hepática cripotgénica se recomienda la determinación de ADN de VHB mediante métodos moleculares de elevada sensibilidad antes de desarrollar signos de cirrosis o CHC [13].

## Conclusiones

La OBI puede persistir durante toda la vida una vez que se ha producido la infección, debido a la larga vida del genoma del VHB, que incluye el ADNccc y el ADN integrado en el genoma del huésped.

Un gran número de pacientes pueden permanecer sin diagnosticar.

La detección de factores de riesgo es importante para prevenir los dos principales problemas asociados a la OBI: la reactivación y la transmisión de VHB.

Es importante el cribado en pacientes con factores de riesgo mediante técnicas moleculares para detección del ADN de VHB, en la medida de lo posible. También deben realizarse ensayos serológicos de VHB que deben incluir la determinación de HBsAg, anti-HBc y anti-HBs.

Existen todavía varios aspectos clínicos y biológicos de la OBI que necesitan ser estudiados con mayor profundidad. También será necesario desarrollar ensayos ultrasensibles de HBsAg estandarizados y validados para la detección en suero de variantes S y que sean capaces de detectar fragmentos de HBsAg, así como ensayos estandarizados y validados para la detección de ADNccc y otras formas de genoma viral de VHB en hígado.
